# How far can you go? Extrapolating values of catalytic activity from known protein landscapes in natural and directed evolution

**DOI:** 10.1039/d5cs01387a

**Published:** 2026-05-13

**Authors:** Douglas B. Kell, Ivayla Roberts

**Affiliations:** a Department of Biochemistry, Cell and Systems Biology, Institute of Systems, Molecular and Integrative Biology, University of Liverpool Crown St Liverpool L69 7ZB UK dbk@liv.ac.uk; b The Novo Nordisk Foundation Centre for Biosustainability, Technical University of Denmark Building 220, Søltofts Plads 200 2800 Kongens Lyngby Denmark; c Department of Physiological Sciences, Faculty of Science, Stellenbosch University Stellenbosch Private Bag X1 Matieland 7602 South Africa

## Abstract

The number of possible variants representing the landscape of a protein sequence of length *N* residues, made of the standard unmodified proteinogenic amino acids, is 20^*N*^; its exhaustive experimental analysis is consequently intractable. Our focus is on the real and perceived shapes of different fitness landscapes. Epistasis refers to a phenomenon by which the ‘best’ amino acid at a given residue depends on the nature of the amino acid at one or more other residues. Because of epistasis, real protein landscapes display peaks representing local maxima in which weak mutation/strong-selection regimes can cause evolution to become trapped, leading to landscapes that are rugged. Fortunately, although they are necessarily somewhat rugged, such protein landscapes possess regularities that admit their modelling from more limited experimental data, using the methods of statistics and machine learning. We provide a variety of arguments that for typical proteins of length 300–500 residues some 10^5^ or 10^6^ examples, and in favourable cases even fewer, are likely sufficient to allow a reasonable initial modelling (and accurate predictive exploration) of the entire 20^*N*^ landscape for properties such as *k*_cat_. The distribution of fitness effects (DFE) around an existing wild type is usually reasonably fitted statistically by a gamma distribution. However, we also survey modern ideas, especially extreme value theory, that allow extrapolation from the known, with a focus on methods – especially the Generalised Pareto Distribution – that provide means for generating the statistical likelihood of obtaining activities or fitnesses far greater than those observed in existing populations as measured with what are small numbers. These likelihoods typically decrease exponentially, as do the decreases in errors as a function of the size of the network and of the training data as found by deep neural network models as ‘universal approximators’. This is entirely consistent with the large differences between the minuscule amount of available sequence-activity data, that are necessarily local in character, reflecting evolutionary contingency, and the overall distribution (20^*N*^, where *N* might usefully be decreased) that would be expected to contain examples that have much better properties than any observed thus far. This consequently requires careful choices of examples drawn from an extensive distribution (using active learning) for predictive modelling. For instance, a widespread view of a trade-off between catalytic activity and thermostability seems to follow directly from inadequate sampling. All of this has significant implications for the understanding, modelling, and optimisation of experiments in directed evolution and the biocatalysts they produce.

## Abstract 2

Rugged protein fitness landscapes can be learned from sparse sequence–activity data. Machine learning plus extreme-value theory can guide directed evolution toward rarer, higher-activity variants.

## Graphical abstract

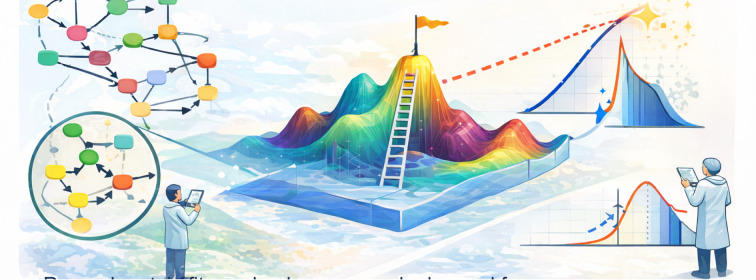

## Introduction

**Fig. 1:**
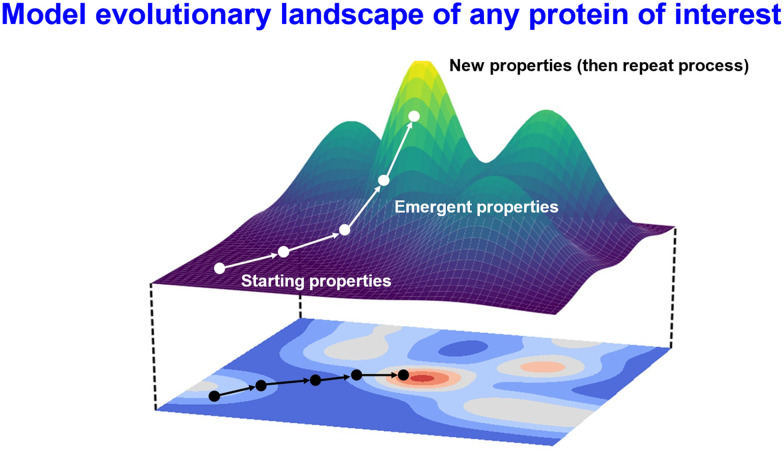
Cartoon illustrating a protein landscape and the evolution of a protein to greater fitnesses as a navigation or adaptive walk across this landscape. Multiple peaks, representing local maxima, are separated by troughs of lower fitness, creating a ‘rugged’ landscape. These features typically arise from epistasis^1^ caused by regimes of weak mutation and strong selection.^22,25,26^ Evidence that peaks are effectively somewhat rounded comes from the diminishing returns during ascent^27,28^ that are usually observed.^29^ The *X* and *Y* axes represent positions in sequence space while the *Z* (height) dimension represents the fitness of interest, *e.g.*, a *k*_cat_ value.

The sequence of a protein determines its activities, and, following Sewall Wright,^1^ variations in sequences and their paired activities (here equated with fitnesses) are usually considered in terms of an evolutionary or fitness landscape (*e.g.*, ref. 2–20). Although the true ‘dimensionality’ of this landscape is that of the protein's length, it is convenient (*e.g.*, ref. 21–24) to visualise these landscapes, just as we do real geographical ones, with the position in sequence space determined by the *X* and *Y* coordinates and the fitness by height (Fig. 1).

As rehearsed in the legend to Fig. 1, the idea of ‘weak mutation/strong selection’^22,25,26^ describes a means of traversal of such fitness landscape in a manner that causes an ever increasing fitness in the desired property. As phrased by Reia and Campos,^30^ “In the simplistic view of evolutionary adaptation, namely strong-selection weak-mutation regime, the conditions *NU* ≪ 1 and *Ns* ≫ 1 hold, where *N* stands for population size, *U* is mutation rate and *s* the selective advantage conferred by the beneficial mutations. Those conditions ensure that selection proceeds much faster than mutations occur. According to this picture, the population is monomorphic most of the time, and the dynamics can be approximated by an adaptive walk, in which the population is depicted as a single entity that moves through the fitness landscape towards fitness peaks”.^30^ The consequence of the existence of such peaks is that populations become stuck in them.

Characterising these landscapes is consequently important if we are to understand them, but there is at once a combinatorial problem:^31^ for a protein of length *N* composed of the 20 common amino acids there are 20^*N*^ possible variants. Just considering ‘local’ variation, the number of alternative variants *M* in a protein of length *N* is given by (*N*!19^*M*^)/(*M*!(*N* − *M*)!).^4,22,32^ For a protein of length 300 residues this equates to 5700 (=(*N* − 1) × *M*), 1·621 × 10^7^ and 31.181 × 10^10^ for just 1, 2 and 3 mutations. Assessing even just the last of these exhaustively would already be seen as technically (and financially) out of reach.

Fig. 1 also illustrates the concept of ruggedness, which relates in general terms to the number and distribution of local maxima separated from each other and from the global maximum by areas or troughs of lower fitness. As with any combinatorial search problem, its ease of solution is effectively determined by some metric of ‘ruggedness’.^33^ A simple landscape, sometimes referred to as the ‘Mount Fuji’ landscape^34^ is defined as follows: “any points on the sequence space have at least one fitter neighbor sequence (an ascending path) which leads to the global optimum in the sequence space in question”.^35^ Thus, there is but a single global maximum, no or few local maxima, and the global maximum can be reached from anywhere just by selecting for increasing fitness; this would clearly be defined as a ‘smooth’ landscape.^36^ At the other end of the ruggedness spectrum would be something pathological (it may be referred to as a ‘bed of nails’ landscape) in which every peak (like a nail) is separated from every other peak by a deep and flat trough that itself gives no information about the potential location of the most adjacent peak(s). In this case an onward evolution to fitter variants would be effectively impossible. While no universal method is optimal for searching all landscapes^37,38^ (but *cf.* ref. 39), we nevertheless recognise that a high-level understanding of ruggedness can aid the selection of suitable algorithms. Unsurprisingly, it is harder to evolve to higher fitnesses by adaptive walks as landscapes become more rugged (*e.g.*, ref. 33 and 40–48).

Note that we here focus mostly on enzymes and binding agents, though the points made apply equally to protein-based biomaterials such as those described in ref. 49–54. We also focus mostly on *k*_cat_, while recognising the importance of other elements of cell-based protein expression such as ensuring solubility (*e.g.*, ref. 55) and the values of secretion (*e.g.*, ref. 56–65). Equivalently, we do not really cover cell-free expression systems (*e.g.*, ref. 66–73), though we recognise their potential benefits when they can be persuaded to work well.

**Fig. 2:**
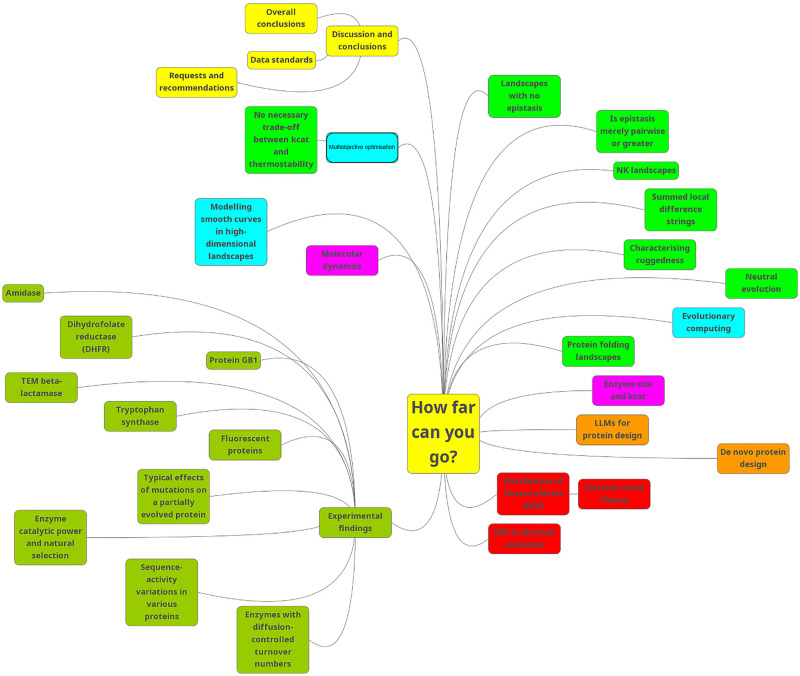
A mind map of the contents of this review. To read this, start at “1 am” and go clockwise.

For convenience, Fig. 2 shows the contents of this review as a Mind Map (see ref. 74 and 75).

### Landscapes with no epistasis

Epistasis describes the idea that the optimum amino acid at a given residue is not independent of the amino acid present in at least one other location.^19,76,77^ It is epistasis that essentially makes the prediction of function from sequence a generally unsolved problem.^78–84^ Reciprocal sign epistasis refers to a circumstance in which two sites distant in sequence space (but in this case close in structural space) need to bind to each other, *e.g.*, a glutamate with a lysine. Thus a mutation at site 1 (from say glutamate to phenylalanine) makes a fitness worse, as does a mutation from a lysine to a phenylalanine at site 2, but mutation of both residues to phenylalanine restores or even enhances the fitness, for inter-residue binding reasons that are rather obvious when one knows the identity of the residues. It is the existence of reciprocal sign epistasis in particular that makes natural landscapes at least somewhat rugged.^19,22,85–87^

The chemical space of small molecules that might harbour bioactivity (*e.g.*, ref. 88–101) is also extremely large (often quoted as 10^60^ molecules or above^102–104^). Consequently, the ‘combinatorial’ search for a ‘good’ protein^31^ is in essence little different from those seeking a ‘good’ small molecule to act as a drug, and ruggedness, activity cliffs^105–110^ (small changes that destroy activity), ‘scaffold hopping’ (*e.g.*, ref. 111–117) (equivalent to large jumps in the landscape), and broad areas of the search space with little or no activity^95,100,118–121^ are common to both. By analogy to the quantitative structure–activity relationship (QSAR) used in pharmacology, Fox and colleagues introduced the idea of ProSAR (protein sequence–activity relationship)^122–127^ to assess experimentally the effect of all possible single mutations on a fitness landscape. If there were no epistasis, evolution to the global maximum would then simply require taking the best amino acid at each residue, thereby reducing the search from 20^*N*^ to 20*N*, effectively mimicking the purest Mt Fuji landscape. Unfortunately for scientists (but fortunately for evolution *via* reasonably stable and locally fit intermediates) biological landscapes do exhibit epistasis,^17,128–131^ for reasons of both structure and biophysics.^19,132–134^ The question is how much, and how does this affect our ability to understand and navigate these landscapes?

### Is epistasis merely pairwise or greater?

The simplest level of epistasis beyond no epistasis is when it occurs between just two residues. So the first important question relates to the extent of epistasis between individual residues, *e.g.*, is just pairwise or is it sometimes three-way or even greater. There is certainly some evidence for higher-order epistases at the organismal level,^81,135–142^ though this is certainly not always the case.^143^ However, for reasons that will become apparent later when we look at how many examples might be needed to model a single protein's landscape reasonably effectively, we will argue that a three-way epistasis can be modelled to a decent approximation as three two-way epistases, so for these purposes we will take it that data from pairwise epistases does allow a suitable approximation, a conclusion also highlighted by Thornton and colleagues.^83^ This leads immediately to the recognition that, for a protein of length *N*, rather than the 20*N* of ProSAR a suitable coverage including all pairwise epistases is given by 20^2^*N*·(*N* − 1)/2, the exponent reflecting the pairwise nature of the epistasis. For a protein of 300 amino acids and for *N* = 20 this amounts to ∼18 M. If we accept that a reasonable landscape might be approximated by five classes (positive, negative, polar, apolar, proline) this drops to 1–2 M. Similarly, structures as estimated computationally will indicate residues that are unlikely to interact, so for modelling purposes *N* can also be decreased significantly, leading to numbers of 10^5^ or even lower. Obviously these kinds of number, though large, are very far below 20^*N*^, and the whole principle of this review is that, while larger populations are inevitably more predictive,^144^ regularities are sufficiently exploitable that we can begin to model large landscapes with surprisingly few examples of mutations.^145,146^ What we are doing here is effectively anchoring points in the landscape for modelling with smooth curves, so these more restricted variants should indeed easily get us down to 10^5^. A significant part of the present analysis is concerned with assessing how good or bad an approximation to the full landscape this is likely to offer.

### Distribution of fitness effects (DFE) and extreme value theory (EVT)

Highly related to the idea of epistatic landscapes, an important reflection of a protein's landscape is the distribution of fitness effects (DFE).^28,147–149^ It effectively describes the size distribution of peaks but without their location (so does not directly need sequence information). As reviewed by ref. 147, the proportion of mutations that are advantageous, effectively neutral and deleterious varies between species (perhaps unsurprisingly) (see also ref. 150), and the DFE also differs between coding and non-coding DNA. The commonest means of fitting such data to a statistical distribution uses the two-parameter gamma distribution family, of which one parameter is the mean while the other is a shape parameter that (as one would expect) determines the shape of the distribution.^147^ Fisher's geometric model^151^ naturally generates fitness epistasis, and the overall level of epistasis can be varied by adjusting the curvature of the rate at which fitness declines with distance from the optimum. A modified gamma distribution is in fact also expected on the basis of Fisher's geometric model,^152,153^ although conversely not all fitness landscapes are compatible with Fisher's geometric model.^154^

In cases where expression levels are varied (*e.g.*, ref. 155), beneficial and deleterious effects are observed in broadly equal measure. By contrast, when genes are deleted completely,^156^ or when mutations are made within an existing (already selected) protein, mutations are more commonly deleterious.^157^ This necessarily follows from the recognition that the ‘wild-type’ starting point has already been subjected to natural selection, and is likely to be in a local maximum as per Fig. 1. The DFE is consequently highly dependent on the extent of epistasis^158^ that thereby governs landscape ruggedness. Other examples that have been fitted to a modified gamma distribution include spontaneous mutations in *Chlamydomonas reinhardtii*^159^ and single mutations in RNA viruses.^160^ A large set of studies is available *via* MaveDB (https://www.mavedb.org).^161,162^

In contrast to deleterious, neutral or weakly beneficial mutations, highly advantageous mutations are not well fitted by the same curve.^163^ The DFE of advantageous mutations seems to be exponential in character, at least for strongly advantageous mutations,^147^ and this is entirely consistent with the expectations of extreme value theory, that we now describe.

### Extreme value theory

Gillespie^164^ was probably the first to use extreme value theory (EVT) for predicting future mutational frequencies for fitter (faster) proteins based on the upper end of existing distributions. We think that EVT is highly appropriate for the problem of present interest (especially where weak mutation and strong selection are involved). EVT has been used to produce estimators for events that are in some sense more extreme or greater than those that have been previously observed, or observed increasingly infrequently as their magnitude increases.^165–169^ Balkema and de Haan^170^ and Pickands^171^ developed the Generalised Pareto distribution for such purposes, and in EVT the upper end of the existing distributions are indeed fit most frequently to the Generalised Pareto distribution.^172–176^ The GDP contains up to three parameters that can be set or optimised (*e.g.*, ref. 177–183), *viz.* the location or threshold *μ*,^179,184^ the scale *σ*, and a shape parameter *ξ*.^185^ In the classic version^165^ (see also ref. 186), EVT recognises that exceedances above a high threshold are more or less well approximated to a generalised Pareto distribution, discusses how the threshold must be “high enough” (often around the 90th–95th percentile of the existing dataset) and develops likelihood-based methods of inference for *σ* and *ξ* once a threshold is chosen. It also discusses how the threshold must be “high enough” for the asymptotics to hold (non-asymptotic strategies are surveyed by Naess^169^). To minimise the inevitable bias-variance dilemma,^179,187^ most commonly (referred to as a conditional GPD) a threshold is first determined and then the other two parameters fitted.^174,188,189^ We have recently used just such a strategy for the prediction of the desirable concentration of a nutraceutical that seems to appear protective against the pregnancy disorder pre-eclampsia.^190^

Among many applications, EVT using the GPD has shown promise in the prediction of drug safety in ever larger populations during the phases of pharmaceutical drug development.^191–193^ Most pertinently, it has been found useful in the prediction of fitnesses beyond an existing distribution,^147,194–198^ where these turn out to be exponentially decreasing in frequency as a function of fitness. As is stands, the availability of large experimental datasets is insufficient to understand where the variation of fitness with position in the landscape transitions from being fitted by a gamma distribution to being fitted by an exponential one, and given that this reflects landscape ruggedness it is likely to vary considerably with the protein of interest.

### DFE in directed evolution

As mentioned above, deep mutational scanning has provided large datasets that (when provided in a usable format – a surprisingly rare event) can be analysed for assessing landscape ruggedness, but they also serve to provide the wherewithal for DFE studies.^6,12,16,199,200^ These provide important data for the understanding of fitness landscapes.

### *NK* landscapes

To seek to model (and learn to navigate) biopolymer landscapes, Kauffman^201–206^ introduced the idea of the *NK* landscape as a tunable fitness landscape in which the size (string or protein length) was determined by *N* and the ruggedness by *K*.^207^ More complex variants (*e.g.*, ref. 208) exist, but the original is still the most widely used. As mentioned in the previous paragraph, natural evolution requires that landscapes be somewhat rugged to preserve local activities in the face of small amounts of mutation,^47^ but not pathologically so as evolution would then be impossible. A *K* of 0 implies something like a Mt Fuji landscape (no epistasis) with increasing values becoming more rugged. In terms of analysing (rather than creating) such landscapes, this is commonly done *via* correlation length or Fourier/amplitude spectra. An exhaustive search of all aptamers of length 10 (so 4^10^ = 1 048 576) able to bind a target protein was consistent with a *K* below 1,^209^ while Aita and colleagues estimated protein folding landscapes to have a *K* in the range 1–3, and Reia and Campos^30^ observed something similar for the fitnesses of Hsp90 and Gb1 in yeast. Some protein landscapes are seen as not excessively rugged (*e.g.*, ref. 143 and 210–213), though other protein landscapes are both expected^26^ and observed (*e.g.*, ref. 17, 19, 85, 131 and 214–221) to be far more rugged, albeit some are easily navigated.^84,212,222^ This implies that predictability requires an appropriate knowledge of the type of landscape involved.

Because actual measurements are comparatively hard and slow,^223^ other authors have used calculations to simulate the fitness of various sequences. du Plessis and colleagues^87^ used free energy calculations, while we^224^ have developed Summed Local Difference Strings that allow for a rugged landscape whose fitness is easily calculated from the sequence of amino acid letters alone.

### Summed local difference strings

Imagine a string in which *A* = 1, *M* = 13 and *Z* = 26, *etc.* One can define, and easily calculate, the ‘fitness’ of a string of such letters by summing the alphabetic distances of adjacent letters.^225^ Thus AZAZAZ is 25 + 25 + 25 + 25 + 25, so the maximum fitness = 25 × (string length −1). AZAZAZ and ZAZAZA thus have the same fitnesses. If we confine ourselves to letters representing the 20 proteinogenic amino acids we can calculate the theoretical maximum fitness of a 100mer (AYAYAY… or YAYAYA.) as 24 × 99 = 2376. For illustrative purposes, we have generated a random string of length 100 (IPTDLWSPFITYSMVNELPWQYDHPKNVSAWHCNDHFWWIQPEFEHMMPTIVNGSKGAVCCNFCHIAIPTWYMTVICNTACRLVCMTQEGLTAVMKQMQN) which has a fitness of 771 and, of course, a search space of 20^100^. Then we randomly mutate between 1 and 100% of the letters and calculate their fitnesses in the same way, as well as the Hamming distance (number of mutated residues) between the starting string and all the others.

**Fig. 3:**
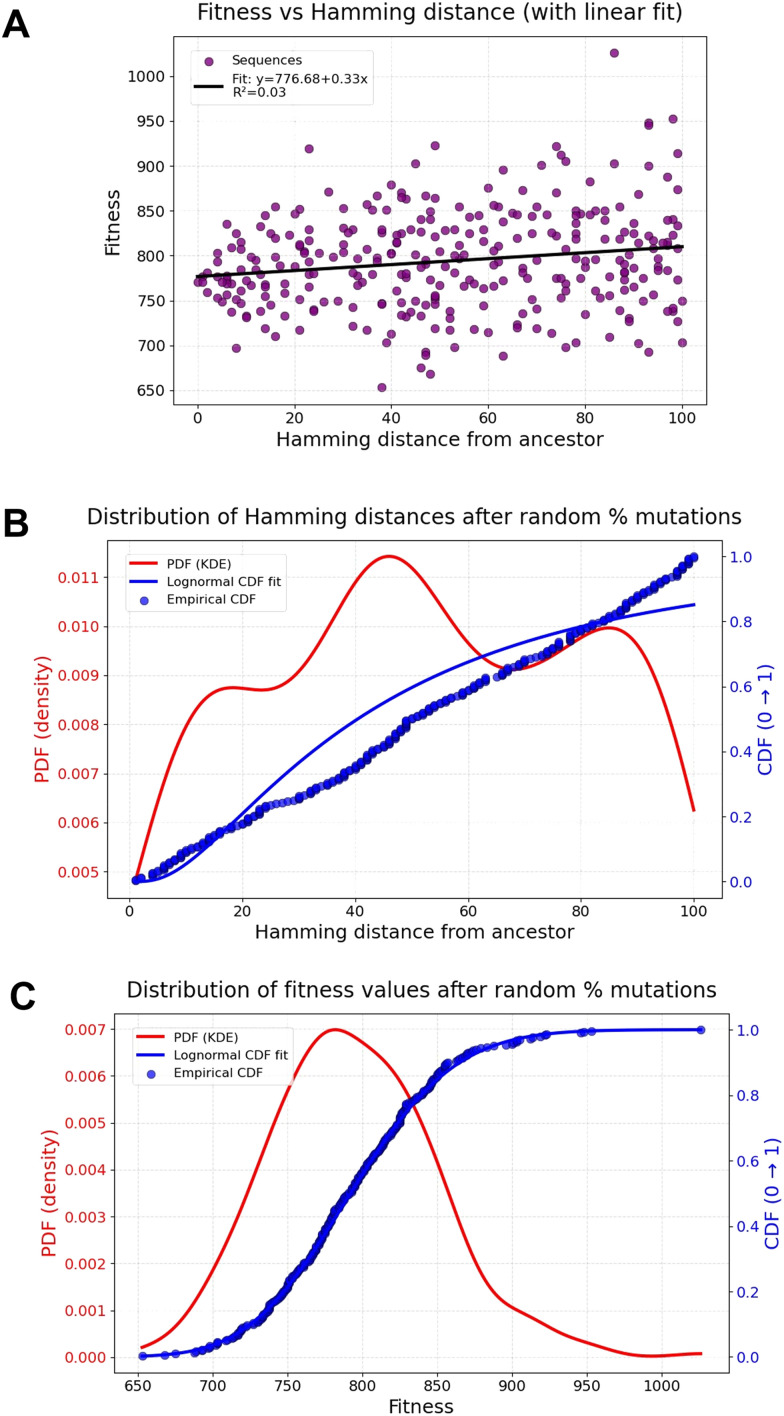
Variation of fitness and Hamming distance from a starting sequence using the summed local distances algorithm when a random number of mutations are introduced into a starting sequence with a fitness of 771 in a landscape in which the maximum fitness is 2376. (A) Lack of fitness-distance correlation relative to the starting sequence, indicating the high overall landscape ruggedness. (B) Distribution of Hamming distances after random mutations. (C) Rarity of high fitnesses when mutating from starting sequences. PDF = probability density function and CDF = cumulative distribution function.

Fig. 3 shows the results of such a simulation with 125 sequences. As expected, there is essentially no correlation between fitness and distance from the starting sequence (Fig. 3A) in what is potentially a rather rugged landscape. However, there is a fairly even spread of the number of mutations (Fig. 3B), as expected, while (Fig. 3C) the probability density function peaks near the starting fitness and, with just 125 examples, finding fitnesses over 950 is a significant rarity (Fig. 2A and C).

**Fig. 4:**
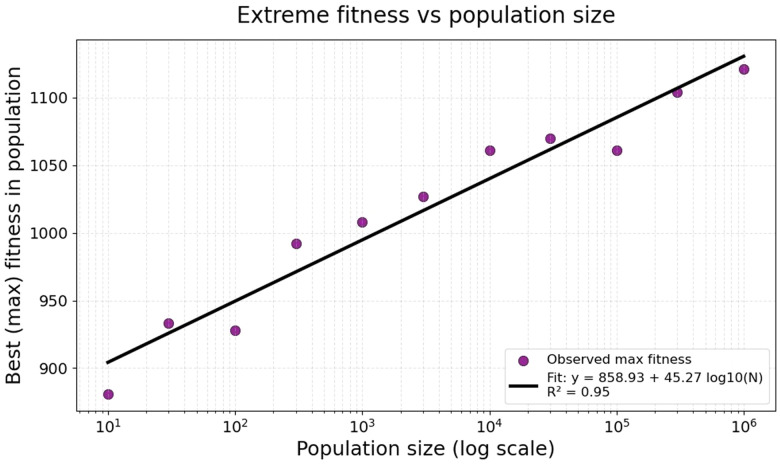
Effect of population size on the best fitness found in a population of random sequences in which the fitness is given by the summed local differences algorithm 132 as described above and in the text.

In a similar vein, Fig. 4 illustrates the fitness of the best mutant, using the same starting sequence, as the population size is varied. Clearly the data are well fitted by an exponential relationship, requiring ever larger populations for each linear increment in fitness. Thus a population size of 10^15^ would have a best fitness, statistically, of just 1533, far below the maximum value. This exponential relationship appears multiple times below, and underscores the need for a good predictive fitness model.

### Characterising ruggedness

Both low sampling^226^ and mistranslation^227^ tend to smooth the peaks (and raise troughs) in epistatic landscapes. Conversely, Song and Zhang^23^ (see also ref. 228) noted that fitness estimation error leads to overestimation of landscape ruggedness. They also rehearse the question of how best to characterise protein landscape ruggedness,^23^ suggesting that four methods are commonly used: (i) the number of fitness peaks in a landscape, (ii) the fraction of pairs of sites displaying reciprocal sign epistasis as defined above, (iii) the roughness to slope ratio (*r*/*s*), which quantifies the extent to which the landscape cannot be described by a linear model where mutations additively determine fitness, and (iv) the fraction of pathways that are blocked, in the sense that a pathway from genotype i to genotype j through single-mutation steps is considered blocked if the fitness is decreased in any of the steps (*i.e.* multiple mutations are required to increase fitness). Each of these methods is effectively a measure of epistasis, and in general, most sensible metrics of ruggedness lead to similar conclusions.^229^ Thus, the choice of method is a matter of taste, software availability, familiarity, and convenience. Another and intuitively obvious method involves fitness-distance correlations^230^ since these will clearly be high on a Mt Fuji landscape and negligible on one like a bed of nails. Note of course that in real landscapes such metrics will vary depending on the starting position in sequence space.

A rather different method is that of Spence and colleagues,^19^ who propose a spectral graph theory approach to measure fitness landscape ruggedness in terms of speed of a heat diffusion analogue.

Early studies that sought to do exhaustive search were necessarily confined to small numbers of residues (*e.g.*, ref. 231), but the availability of cheap(er) sequencing (sometimes referred to as deep mutational scanning (*e.g.*, ref. 6, 12, 16, 18, 145, 200 and 232–248) has brought much more extensive analyses into play (*e.g.*, ref. 10, 30 and 249–251 and see later).

### Neutral evolution

One way to escape a local peak in the face of weak mutation-strong selection is by so-called neutral mutations (*e.g.*, ref. 252–258), in which one finds routes that involve only small losses in fitness (relative to population size and selection pressure) *en route* to higher fitness peaks^6^, and neutral genetic drift has been considered useful for the purposes of directed evolution (*e.g.*, ref. 259–268). This said, the great advantage of directed over natural evolution is that one can choose how to override the weak-mutation-strong-selection property of natural evolution in small, bounded populations, and we would argue that neutral evolution is not going to be of such major importance in modern directed evolution (where one has reasonably abundant sequence-activity data) if one has developed an understanding of the landscape that can effectively permit the equivalent of ‘scaffold hopping’.

### A note on evolutionary computing

Evolutionary computing describes a series of methods for addressing combinatorial search problems that are based on principles very similar to those of natural evolution.^269^ It is used explicitly to model, understand and navigate fitness landscapes of the type illustrated in Fig. 1. Here the individuals in the population are candidate solutions to a problem of interest, and they can be evolved by processes akin to mutation, recombination and selection. These methods can be highly efficient, and have been used to provide solutions for very high-dimensional problems that are seen as close to the global optimum. Evolutionary computing is not of recent origin and has proved of value in metabolic engineering^270–273^), and the following references may usefully be consulted.^274–296^ Evolutionary computing also admits variations of mutation and recombination (such as uniform crossover^297^) that are not likely to be exploited in natural evolution.

A particularly important lesson from studies of evolutionary computing is that it is vital to maintain diversity in the evolving population. Simply selecting the best individual in each round effectively ensures that one will soon become trapped in a local peak from which it is effectively impossible to escape. This was regularly found in the early literature of directed evolution when cheap gene sequencing was not available, and can clearly be demonstrated when it is.^298^

Another issue in the variant of evolutionary computing known as genetic programming is referred to as ‘bloat’.^299^ ‘Bloat’ describes how the selection for improved activities is more easily done by making entities larger, in that making them smaller is usually associated locally with a lowered fitness and such variants are then selected out.

Evolutionary computing is also relevant to the question of active learning.^18,84,300–303^ For the present problem, this describes the idea that, armed with a model of a landscape, rather than picking a random sequence and asking what the paired fitness would be one can choose more rationally which sequences to interrogate the model with, thereby both exploiting, and increasing one's knowledge of, the better parts of the landscape.^14^ A classic article^304^ uses what amount to Bayesian methods that combine areas of the search space that seem promising with those that are seen as underexplored (or have greater uncertainty^305^), thus seeking simultaneously to improve both the objective function and the model of the landscape. Evolutionary computing methods are ideally suited for this purpose. (They can also be used to train neural networks, as optimising weights and architectures is effectively a combinatorial search problem too; this is known as neuroevolution.^306–312^)

### The relationship of protein folding landscapes to the directed evolution problem

Protein folding provides another example in which there is a massive search space in that if each amino acid in a protein of *N* residues could adopt (for example) 10 conformations the number of conformations is 10^*N*^, which again cannot possibly be explored.^313–315^ It maps precisely onto the kind of landscape navigation problem that is our focus. The solution to this apparent paradox^313^ is of course that they are not explored, and that (modulo chaperone proteins) proteins use both the sequence itself^316,317^ and favoured kinetic pathways to fold up into stable structures as they emerge from the ribosome. These structures are not in fact the thermodynamically most stable (as was once assumed), and most proteins (see an analysis using AmyloGram^318^ for all human proteins at^319^) are capable of adopting so-called ‘amyloid’ forms with crossed-β motifs that are significantly more stable^320^. However, the usual state is separated from the amyloid form by high energy barriers, of maybe 36–38 kJ mol^−1^ in the case of prions.^321^

**Fig. 5:**
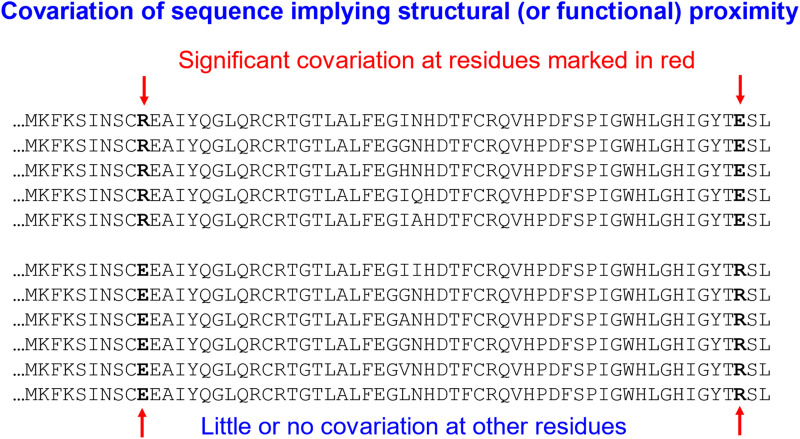
The use of covariance analysis to provide a spatial constraint in *de novo* protein folding studies. See text for references and discussion.

Before the arrival of Alphafold,^322–328^ Rosettafold^329,330^ and similar, a significant advance was made in the *de novo* protein folding field through covariance analysis of series of sequences.^331–338^ Thus, if at a certain position there were multiple sequences that contained a negatively charge residue (glu or asp), while at another residue there was always a positive charge (lys or arg), but that for other orthologues the charges were reversed (so they always covaried), it could be assumed that these residues were in close proximity to each other in the 3D structure (also providing another clear example of reciprocal sign epistasis) (Fig. 5).

Using these interactions estimated from covariance analysis as anchors served to constrain the protein folding problem so significantly that with 100 000 examples or so, it was possible to fold many proteins to within ∼4 Å of their correct values. In a sense this is a problem isomorphous to that of present interest, merely replacing an activity landscape by a folding landscape. Its importance for us is in the recognition (and see below) that ‘only’ some 10^5^ examples or so were sufficient to model the (protein folding) landscape rather well.

### Enzyme size and *k*_cat_

Eukaryotic proteins have an average size of 472 amino acid residues, whereas bacterial (320 residues) and archaeal proteins (283 residues) are typically smaller,^339^ but still large. For a given yield of recombinant protein a smaller enzyme with a given *k*_cat_ obviously admits a greater activity, and is correspondingly easier to improve using directed evolution. Consequently, an often-asked question is “why are natural enzymes so big?” (*e.g.*, ref. 340 and 341). A common answer (*e.g.*, ref. 342) would be that this is necessary to form the 3D conformations that the active site requires for catalytic activity, but as well as being a self-defining prophecy this does not alone seem a satisfactory answer. Another notes a relationship between the molecular mass and the lowering of the activation energy for a reaction^341^ but again no systematic studies of homologues seem to have been performed. A third is related to the phenomenon of ‘bloat’ as described in the section on evolutionary computing; increasing a protein's size in a local landscape is far less likely to cause a significant decrease in fitness than removing residues that potentially mess up the structure considerably. Consequently, the question of (changes in) size is itself highly relevant to the understanding of fitness landscapes. Commonly, organisms sought to solve this fitness issue by gene duplication;^343,344^ one could evolve a separate gene while retaining a sufficient activity based on the retention of activity in the first gene.^345^ ‘Contingency’ or ‘historical contingency’^346,347^ decribes the fact that how evolution proceeds depends on earlier events, and not least since mutation is largely random evolution cannot be taken to be deterministic. Overall, then, we consider that certain sequences simply got fixed in inadequate maxima as a result of evolutionary contingency, and that large sizes are not *de rigeur*, for at least three classes of reason.

First, some enzymes actually are very small. This said, the few enzymes under 10 kDa (*ca.* 90 amino acid residues) mostly remain poorly characterized.^348^ In addition, we know of course from any number of directed evolution studies that enzymes can be made much faster without getting bigger (∼1000-fold faster in one case – lovD – in which changes in the ground-state structure of the enzyme were undetectable^349^). The counterfactual is therefore that small(er) enzymes might be created/evolved to be just as fast as those produced by natural or directed evolution. While organocatalysis can be effected with a single amino acid (especially proline),^350–353^ we are here interested in polypeptides.^354^ The record small subunit is seemingly 62 amino acids (aa) for (the hexameric and highly active) 4-oxalocrotonate tautomerase^355^ and there is also a 6 kDa metalloprotease.^356^ As stated by ref. 357, “Two esterase enzymes have been isolated, one from *Candida lipolytica* and one from *Bacillus stearothermophilus*, which are characterised by an unusually small molecular weight. The *Candida* enzyme is 5.7 kDa, with 56 amino acid residues and the *Bacillus* enzyme is 1.57 kDa,^358^ with only 17 residues. It is also more thermostable. Both are metalloenzymes.” Similarly sized enzymes have been observed in fungi,^359^ which the authors refer to as microenzymes (a term possibly coined by ref. 360).

Other small enzymes include an ascorbate oxidase in barley with MW <10 kDa,^361^ a urease with 97 residues,^362^ the 74-residue ‘AlleyCat’ haem enzymes that can effect Kemp elimination^363^ (see also ref. 364–367), and a 98-mer acylphosphatase.^368,369^ Certainly ‘miniproteins’ of 40–50 residues do fold,^370^ and even a 29-mer can exhibit some reasonable catalytic activity.^371–373^

Secondly, the famously fast^374,375^ triose phosphate isomerase is normally a dimer, with a monomeric mutant having very low rates.^376,377^ However, directed evolution can be used to improve these,^378^ albeit not to wild-type levels.

Thirdly, there are a great many examples of convergent evolution in which entirely non-homologous proteins catalyse the same reaction,^379–383^ strongly implying the role of contingency in natural evolution (note that convergent evolution is not inevitable; many solute carriers, for instance, have evolved by divergent evolution^384–388^ by which a basic scaffold was found useful for a specific purpose, *i.e.* transmembrane transport, and modified to deal with different substrates).

All of the above speaks to the importance of contingency in natural evolution (*e.g.*, ref. 28, 346 and 389–391), consistent with the ideas that we stress here of weak mutation and strong selection creating epistatic, rugged landscapes from whose peaks it is relatively hard (under these conditions) to escape.

### Large language models (LLMs) for protein design

Large language models (LLMs) have come to dominate modern text-based AI and machine learning methods. They are trained on large sequences of words and learn to predict, statistically, the next letter or word (‘token’) in a sequence. The transformer architecture^392^ is the overwhelmingly dominant architecture used (it can be applied to many other problems, including predicting the structure of small molecules from their mass spectra^393^). Since protein design is such an important problem^82^, many LLMs for protein design have come to the fore in the public domain (and many others are proprietary). This purely computational protein design approach is a field that is very fast moving such that any survey will soon be superseded; however, we do recognise that it is highly germane to the general problem of protein landscape simulation, understanding, and even creation, so we provide a listing in Table 1. This is also the place to note the potential for biosecurity concerns that are now beginning to be addressed more openly.^394^

**Table 1:** A sample of large language and related AI models as applied to antibody or enzyme design

Model	Comments	Selected ref.
Anon (not named)	Early LLM including function	395
AntiFold	Antibody-specific inverse folding LLM, fine-tuned from ESM-IF1^396^	397
BOM-POOLING	Optimising representations when input lengths vary	398
CrossDesign	Enzyme design under low-data regimes	399
ESM-IF1-combo	Design pipeline using Foldseek ESM-IF1 + AlphaFold2 for peptide binders to targets.	400
ESM3	Combines Rosetta Sequence Design with protein language model predictions using evolutionary scale modeling (ESM) as a restraint	401 and 402
EVOLVEpro	Improved six proteins, including T7 RNA polymerase and a serine integrase	403
GENzyme	Reaction-driven enzyme design	404
IgHuAb	LLM-generated human antibody library (SynAbLib)	405
IgLM	Infilling language modelling for antibody sequence design; uses masked infilling	406 and 407
METL	Combines thermostability and protein engineering	408
MIF/MIF-ST	Structure-conditioned masked LM; masking improves inverse folding & design scoring	409
MSA transformer	Uses a masked LM to generate *de novo* sequences from a multiple sequence alignment transformer	200 and 410
p-IgGen	LLM for paired heavy–light chain antibody generation with desirable biophysical properties	411
PLMFit	Useful benchmarking study of ESM2, ProGen2, and ProteinBert	412
Pool PaRTI	Focus on variable sequence lengths	413
PRIME	Focus on thermostability	414
ProDualNet	Combines a protein language model with a structure model to co-design binders for dual targets	415
ProGen	Conditional LLM for *de novo* sequence generation with functional activity across families with sequence homologies as low as 31.4%	416
ProGen2	Open-sourced 6.4Bn-parameter upscale of ProGen; improved zero-shot fitness and controllable generation	407 and 417
ProstT5	ProtT5-derived bilingual Language model; large speed-up on AlphaFold to aid structure-aware design	418
Protein-as-Second-Language	Claims to avoid the notorious quadratic problem of standard transformers	419
ProteinBERT	Early BERT-style protein language model; has been widely fine-tuned for function and property prediction supporting design	420
ProteinGenerator	Not really an LLM as it uses denoising diffusion in sequence space	330
ProteinMPNN	A graph neural network rather than an LLM, for inverse folding	421
ProtFlash	Claims linear complexity by using a mixed chunk attention mechanism	422
ProtGPT2	Autoregressive LLM for *de novo* sequence generation; samples unexplored regions of protein space	423
Reviews		416 and 424–428
RFdiffusion	Another based on diffusion over structures (not an LLM), in combination with RosettaFold	429
RFDiffusion2	Updated version of the above	430
RiffDiff	A hybrid machine learning and atomistic modelling strategy for scaffolding catalytic arrays in *de novo* proteins	431
SSRL	Structure-guided sequence representation learning	432
Unirep	Deep learning from sequence alone	433 and 434

Many individual steps in synthetic biology have now been automated (including those in our own work, *e.g.*, ref. 435–440). However, it is to be stressed that these AI-based methods^441^ are increasingly being accompanied by a fully closed-loop^442–444^ form of automation^445–449^ of the entire Design-Build-Test-Learn cycle of synthetic biology.

### *De novo* fold and protein design

Much of the ethos of this review is based around the combinatorial fact that neither experimenters nor natural evolution have sampled anything other than a tiny fraction of possible sequence space, even for small proteins. In terms of protein folds, one may suppose that the more examples we can sample the more we shall find.^450^ Databases such as CATH^451^ have over 2000 folds (significantly increased by the addition of AlphaFold-predicted structures^452^) and hundreds of millions of domains.^453^ In addition, we note the significance of metamorphic^454–457^ or ‘fold-switching’^458–460^ proteins that can mediate major evolutionary transitions in scaffolds,^461^ while the existence of amyloidogenic proteins reflects the fact that the commonest structures as formed by the ribosome are not thermodynamically the most stable.^462–465^

All of this said, the same issues we have highlighted, of weak mutation, strong selection, contingency,^390^ and epistasis, imply that natural evolution has sampled only a tiny fraction of possible folds,^466^ and there is now considerable interest, and some notable success, in designing completely novel folds, structures and functions *de novo*, leading to stable, soluble proteins with topologies absent from current structure databases (*e.g.*, ref. 429 and 467–482). This very much highlights the importance of understanding protein sequence/activity landscapes.

### Experimental findings

#### Enzymes with diffusion-controlled turnover numbers

With rare exceptions (*e.g.*, ref. 483 and 484; Table 2) the maximum turnover numbers of enzymes are normally very far below the diffusion controlled limit, presumably (see below) because there was little selection pressure to raise them.

**Table 2:** Enzymes whose activity is considered to be at or near the diffusion-controlled limit

Enzyme	Comments	Selected ref.
Acetylcholinesterase	*k*_cat_/*K*_m_ > 10^8^ M^−1^ s^−1^	485 and 486
Carbonic anhydrase	*k*_cat_/*K*_m_ ∼ 10^9^ M^−1^ s^−1^	487
Catalase	*k*_cat_/*K*_m_ > 10^7^ M^−1^ s^−1^	488 and 489
Superoxide dismutase (Mn)	*k*_cat_/*K*_m_ ∼ 8.10^8^ M^−1^ s^−1^	490
Triose phosphate isomerase	The original classic; *k*_cat_/*K*_m_ > 10^8^ M^−1^ s^−1^	374, 375, 483 and 484

While the reactions catalysed by these enzymes are relatively simple, it is not obvious for chemical reasons why virtually any enzyme could not be able to attain such rates, and we believe that in most cases they will be able to. Again, the simplest explanation (additional to lack of selection pressure) is that most proteins got stuck in areas of the landscape from which they could not simply escape.

### Sequence-activity variations in various proteins

**Fig. 6:**
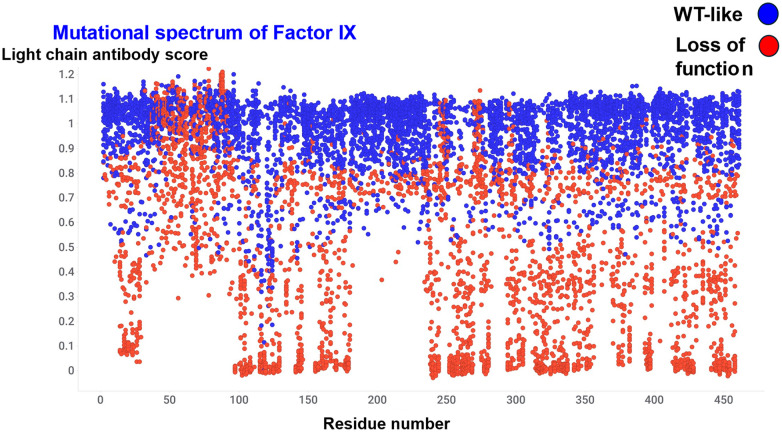
Mutational spectrum of Factor IX. Data taken from ref. 491.

We next look at some experimental datasets that give an indication of the kinds of variation in (sequence and) activity that may be observed in real proteins. Obviously we are here starting with proteins that have themselves been selected (and likely been trapped in local fitness peaks) during natural evolution, so we are not modelling the very earliest stages in an evolution. We begin with an enzyme but look at the binding of an antibody thereto as an indication (in this case) of misfolding or effectiveness of secretion. Fowler and colleagues^491^ studied 44 816 variant effects for 436 synonymous variants and 8528 of the 8759 possible missense variants of the serine protease (clotting) Factor IX (FIX). Their Table S4 contains 8964 examples, of which 5085 were encoded as wild type and 3879 as loss of function. We have plotted these data as a function of residue number in Fig. 6. This allows us to make the following observations, that are fairly generally true. First, almost no residue is completely immune from causing trouble (loss of function) when mutated, though some areas (such as the residues from ∼180 to ∼230) are much more resilient than others (see also ref. 134). For instance, surface residue are much more tolerant than are buried ones.^199,492^ Secondly, most mutations are deleterious (see also ref. 160, 493 and 494).

**Fig. 7:**
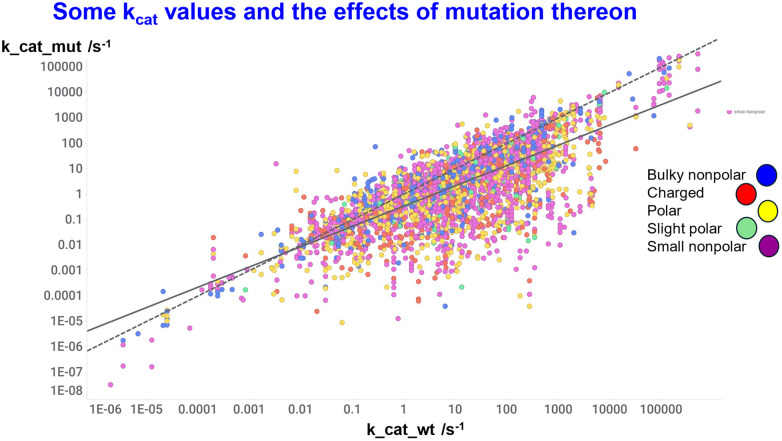
Data from Yan *et al.*^495^ on a large series of enzymes and mutants thereof. Continuous line is line of best fit, while dotted line = line of identity.

Fig. 7 shows data^495^ on a large series of enzymes, where it is clear from the relative slopes of the line and best fit (continuous line) and the line of identity (dotted line) that mutations from better starting points tend to be deleterious, effectively reflecting peak in the system.

### Enzyme catalytic power and natural selection

**Fig. 8:**
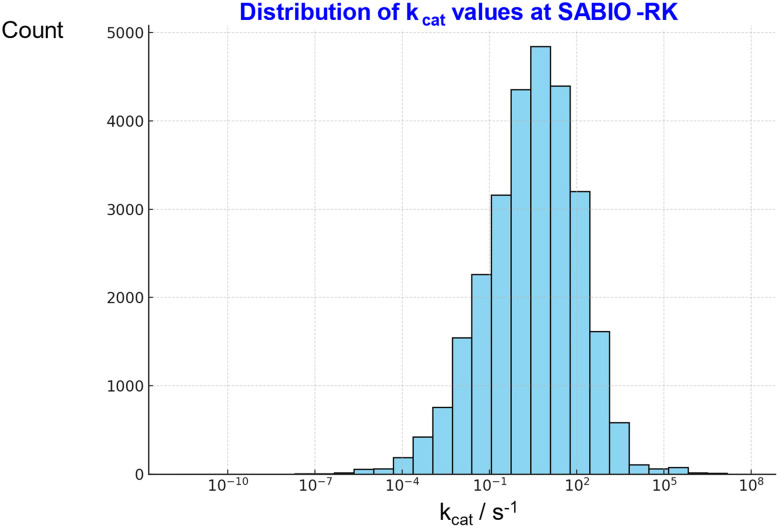
Distribution of 27 757 ‘starting’ values of *k*_cat_ as downloaded from SABIO-RK.

The usual metric for enzyme catalytic power is *k*_cat_/*K*_m_. In the more common Michaelis–Menten kinetics,^496–499^ diffusion of substrate to the enzyme active site is fast relative to the catalytic event^500^ (but see ref. 501). Diffusion-controlled enzymes, which it has been claimed can have catalytic powers as great as 10^10^ M^−1^ s^−1^, ref. 502 (10^8−9^ M^−1^ s^−1^ is more commonly quoted) tend to exhibit Briggs–Haldane kinetics but are rare. Most values of catalytic power are far lower than this. In the case of directed evolution for industrial enzymes we normally have substrate concentrations far above most values of *K*_m_, and we are really more interested in optimising values of *k*_cat_ or turnover number (that with stability determines space-time yield^503–507^), not least to avoid the burden of increasing protein synthesis.^26,508–512^ Protein levels even for a given amino acid sequence also depend strongly on codon usage, especially *via* RNA stability,^513^ as well as the thermodynamics of the reaction involved.^514,515^ Few of these enzyme turnover numbers naturally exceed 100 s^−1^, ref. 516 (the median for natural enzymes is ∼10 s^−1^, ref. 517), and in directed evolution most are far below this^504,518,519^. For instance the storied sitagliptin example^520^ improved an enzyme many thousandfold but the final *k*_cat_ was still only ∼25 s^−1^. Tables of *k*_cat_, *k*_cat_/*K*_m_*etc.* are available at a variety of databases^521^ such as BRENDA^522^ and SABIO-RK.^523,524^ A distillation of the latter is given in Fig. 8.

Because extensive measurements covering many residues are often quite poorly available,^18,223^ it is increasingly possible to use the methods of machine learning to calculate *k*_cat_ and other enzyme kinetic parameter values *de novo*.^519,525–537^ This will be very valuable for both creating and navigating activity landscapes.

Metabolic control analysis^538–541^ is a method of local sensitivity analysis^542^ in which the normalised effect of a small change in enzyme activity or concentration d*E*/*E* creates a normalised change in flux d*J*/*J*. Their ratio (d*J*/*J*)/(d*E*/*E*) is known as the flux-control coefficient and, importantly, the sum of the flux-control coefficients for a flux of interest, over all the enzymes in a system adds to 1. Consequently most flux-control coefficients have small values and increasing the rate of an individual enzyme simply means that it contributes increasingly less to flux control. Consequently, because (or when) they are embedded in metabolic networks, there is little or no selection pressure in natural evolution for individual enzymes to seek to achieve unusually high values of *k*_cat_. This is sufficient to explain why most mutations in diploid systems are recessive.^345^

Whether the chemistry of any enzymatic reaction (or at least most) could be converted to run at near diffusion-controlled rates is an open question, but our prejudice is that if we understood landscapes well enough there is no *a priori* reason why not. As stated above, the modest values for *k*_cat_ seen in natural enzymes are easily seen to result from a combination of a lack of selection and the combination of weak mutation, strong selection and epistasis trapping enzymes in local optima that are far from the maximum. We give further arguments below based on the similarity of the exponential distributions of both extreme value theory and neural network scaling.

### Typical effects of mutations on a partially evolved protein

Given that a starting protein of interest is some kind of wild type that has presumably evolved to a local maximum it is unsurprising that most initial mutations (commonly about one half^434,495^) result in lower fitnesses, meaning that one has to seek multiple mutations to find a better peak (‘reculer pour mieux sauter’^543^).^544^ The default view might be that if half of the single mutations are deleterious then only a quarter of double mutations and 1/(2^*n*^) of *n* mutations might be better. Fortunately this dismal view fails to take into account the existence of reciprocal sign epistasis and also that the improvements may be much better than this exponential relation would indicate.

### Fluorescent proteins

Fluorescence is a property that is easily measured, and the classical Green Fluorescent Protein (GFP) from *Aequorea victoria*, with just ∼250 residues, provides the archetype.^545,546^ Sarkisyan *et al*.^547^ studied the GFP landscape, finding that the effect of mutations on fluorescence was positively correlated with site conservation. They also commented that “The broad congruence of our data with the prevalence of epistasis from long-term evolution suggests that the shape of the local fitness landscape can be extrapolated to a larger scale”^547^. This challenge was effectively taken up in an important paper by Biswas *et al*.^434^ In this work, they studied both GFP and β-lactamase variants for which they had nearly 10^5^ functional sequences. Neural networks require numerical rather than sequence inputs,^548^ so their own LLM UniRep was used to form a numerical representation, an embedding that effectively learned secondary structures from primary sequences. A supervised method was used to fine tune this representation, and the model landscape could then be navigated to produce variants with both high novelty in sequence space and high activity. This provided an important indication that it was indeed possible to generalise from landscapes trained with numbers far smaller than 10^*N*^. The same was true for TEM1 β-lactamase,^434^ and Wagner^84^ considers (with evidence) that this is likely to be generally true.

**Fig. 9:**
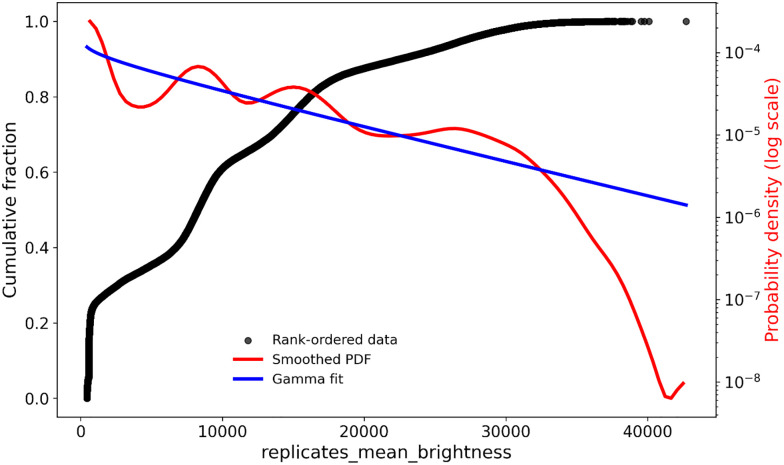
Probability density function, gamma distribution and cumulative distribution function of 93 925 variants of green fluorescent protein studied by Gonzalez Somermeyer and colleagues.^218^.

An especially large dataset on GFP and related proteins was provided by Gonzalez Somermeyer and colleagues.^218^ It consisted of some 93 925 variants with attendant fitnesses. Data were obtained from their supplementary information (SI). These are plotted in Fig. 9 as probability density (PDF) and cumulative (CDF) distributions, along with the fit to a gamma distribution. An interesting feature of this dataset, given its starting points in the GFP of four organisms, three with 18%, 59%, and 82% sequence divergence from the classical (*Aequorea victoria*) GFP, was the existence of four major fitness peaks, two sharp and two flatter, and these are evident in both the PDF and CDF of Fig. 9. Of especial interest in the context of this review is the fact that above a brightness of some 30 000 or so the distribution of fitnesses evidently turns from something like a gamma distribution to an exponential one, an argument that is a central feature of this review.

### TEM1 β-lactamase

**Fig. 10:**
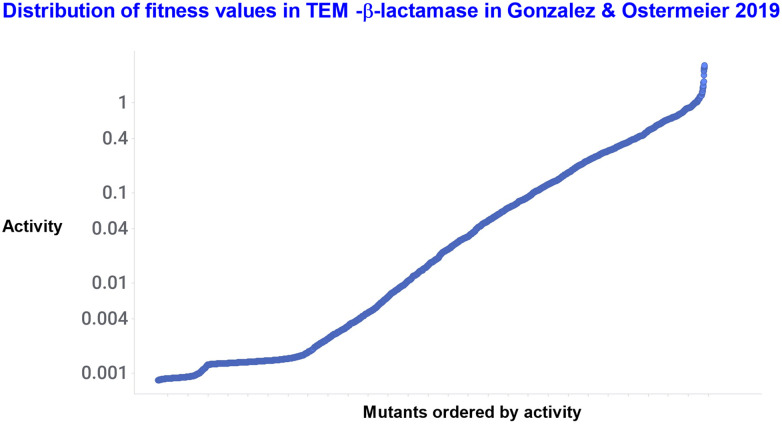
Distribution of activities in 4507 mutants of TEM-β-lactamase. Data are taken from the SI of ref. 549.

Gonzalez and Ostermeier^549^ also studied intragenic epistasis among several thousand pairs of mutations in adjacent residues (‘sequential mutations’) in TEM-1 β-lactamase. Negative epistasis (52%) occurred 7.6 times as frequently as did positive epistasis (6.8%). The distribution of activities is shown in Fig. 10, where the activity levels cover more than three orders of magnitude.

**Fig. 11:**
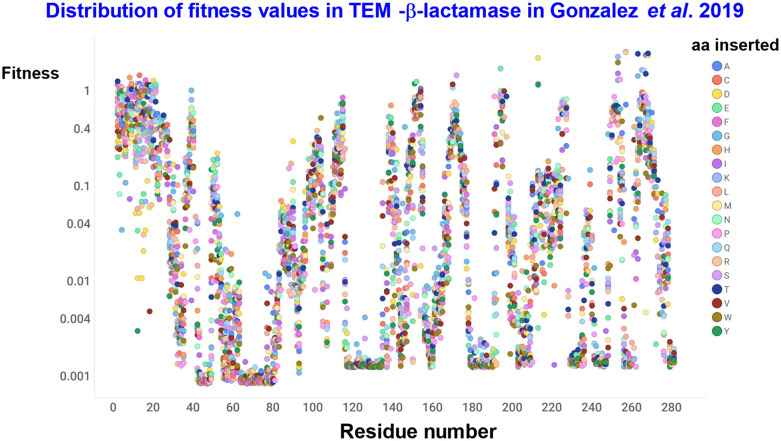
Effect of insertions on the fitness levels of TEM-β-lactamase. Data are taken from the SI of ref. 550.

The same group^550^ then went on to study the effect of single indels, with the results for insertions shown in Fig. 11. Again the range covered more than three orders of magnitude, while insertions tended to be somewhat less deleterious than deletions, and the largest effects were seen in regions with significant secondary structure. Over half of insertions (51%) and deletions (59%) resulted in at least a 100-fold decrease in fitness relative to TEM-1. 9.8% of insertions and 11% of deletions retained 50% of wild-type fitness, although 40.9% of these were in the signal sequence (the first 23 aa), probably reflecting varying expression levels more than *k*_cat_ changes. In a similar study, Macdonald and colleagues also found that deletions were most disruptive overall, that beta sheets are most sensitive to indels, and flexible loops are sensitive to deletions yet tolerate insertions.^551^ In general, disruptiveness depends on the local structural context;^552^ for instance, prolines are not tolerated inside α-helices.^553^

### Tryptophan synthase

Johnston *et al*.^251^ performed an exhaustive analysis of the effects of changing four amino acids (20^4^ = 160 000) in the active site of the beta subunit of a thermostable tryptophan synthase (TrpB). Jason Yang kindly provided the data *via*https://data.caltech.edu/records/h5rah-5z170. Just 10 140 of the mutants were seen as active. Interestingly, the most-fit TrpB variants contained a substitution (K227) that is nearly absent in natural TrpB sequences, which could be seen as consistent with the view that once activity is sufficient for the needs of the organism in its environments there is little selection pressure to increase it.

**Fig. 12:**
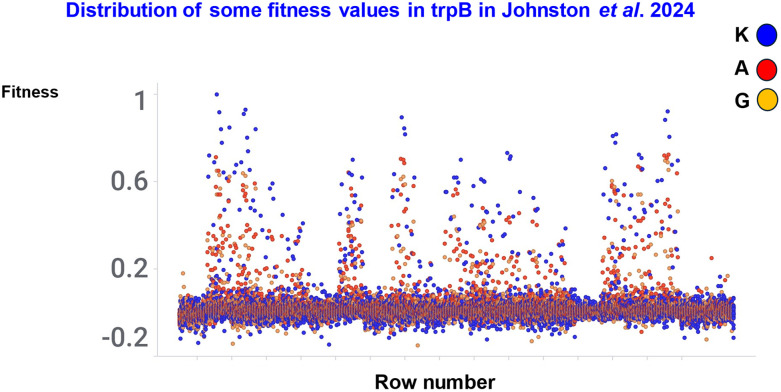
Illustration of the high fitness value of K227 (compared with A227 and G227) in some contexts in trpB. Data from ref. 251.

This is illustrated in Fig. 12 in terms of the fitness of three of the amino acids at position 227. Clearly (as stated) K227 is the highest (other amino acids are excluded for clarity) but equally clearly there are other contexts in which K227 is very poor indeed, showing again how very epistatic is this particular landscape. Of course we do need to stress again that the starting sequence is a wild type that has been selected by natural evolution, albeit in a thermophile rather than the mesophilic recombinant host used (*E. coli*), and is to be seen as existing in some kind of a local peak, so it is not surprising that most mutations are less fit than the average.

**Fig. 13:**
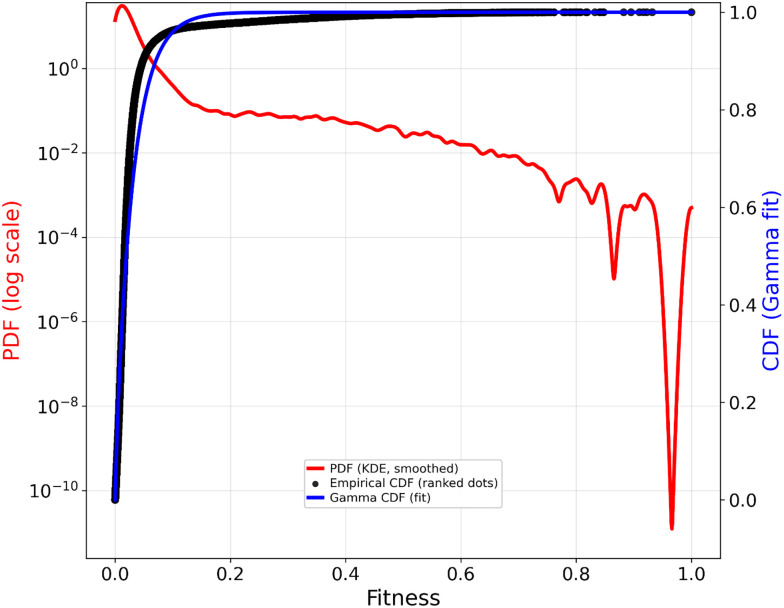
Probability distribution function (PDF) and fit to a gamma distribution function for the cumulative fitnesses in the large dataset of Johnston *et al*.^251^ on trpB.

In a similar vein, Fig. 13 shows the fitness and its probability density distribution for this large dataset^251^, indicating how most mutations are less fit, but that the cumulative distribution function is tolerably fitted by a gamma function (whose parameters are shape = 0.869 and scale (*θ*) = 0.034). Although the larger fitnesses are increasingly few in number, it is reasonable to state that above a fitness of about 0.5 there is a change in slope and these follow an exponential distribution as stressed throughout this review.

### Protein GB1

Wu *et al*. performed a similar exhaustive study at 4 sites (V39, D40, G41, V54; 160 000 variants) in the 56-amino acid B1 domain of protein G, an immunoglobulin-binding protein expressed in certain streptococci. As expected, most mutants had a lower fitness compared to the wild type (VDGV, whose fitness was normalised to 1), 2.4% of mutants were beneficial. The best (FWAA) was nearly 9 times fitter.

**Fig. 14:**
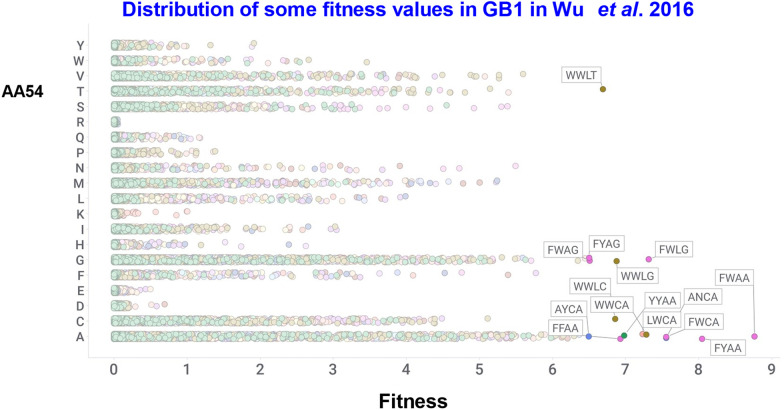
Fitness values for various mutations in AA54 of GB1,^10^ coloured by the residue at AA39. The top 15 sequences are labelled.

**Fig. 15:**
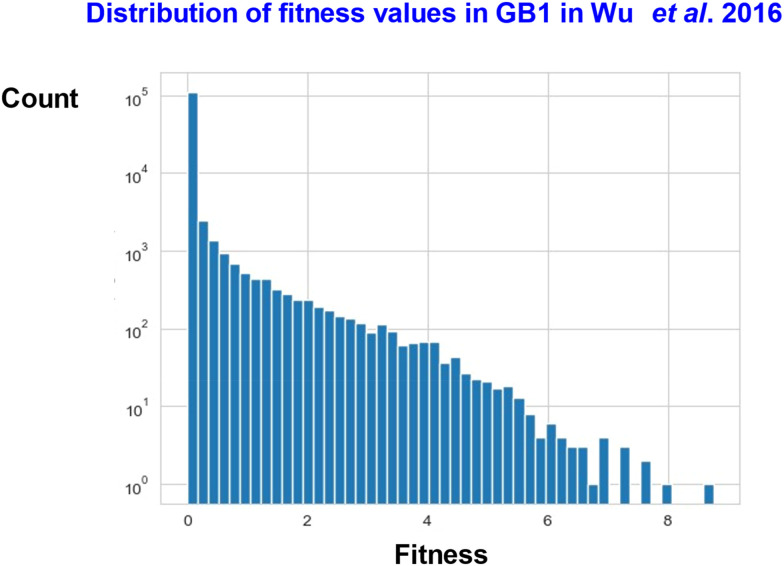
Distribution of fitness values as taken from the data in Table S4 of Wu *et al*.^10^

Fig. 14 and 15 show some of the data (taken from Table S4 of ref. 10).

These figures serve to illustrate several points. First, that the best 15 variants do not share a single residue in common with the VDGV wild type (see also ref. 554). Secondly, for D40 and V54 they are not even of the same classes (*i.e.* negatively charged and hydrophobic) as that of the original, and thirdly, above the wild-type fitness of 1 the frequency is evidently decreasing exponentially with increasing fitness.

### Dihydrofolate reductase (DHFR)

D’Costa and colleagues performed a detailed study^16^ of the protein fitness landscape of DHFR, an enzyme whose ‘fitness’ can be both selected for and assessed in terms of resistance to trimethoprim. They found extensive epistasis but an overall global peak that was evolutionarily accessible from most starting sequences. In a similar vein, Papkou, Wagner and colleagues^212^ created a large biological fitness landscape (>260 000 mutants) using CRISPR-Cas9 gene editing of the *Escherichia coli* dihydrofolate reductase, fitness again being assessed as growth rates in the presence of trimethoprim. In this case just 17 774 of the strains (6.8%) were viable, encoding 1630 unique amino acid sequences.^84^ In this latter paper, 90% of the predictive power could be attained with just 7.8% (1400) of the genotypes.

### Amidase

Wrenbeck *et al*.^555^ analysed over 7000 mutants of an amidase, representing over 96% of non-synonymous single mutations, for their activity against three different amides, with the distribution of beneficial mutations being quite different for each substrate while seen as ‘exponential’.

### The role of (molecular) dynamics in enzyme landscapes

Improvements in enzymatic rate constants normally occur far from the active site, and we have argued^22^ that this is because of the important role of dynamics in enzyme catalysis. Specifically, it is recognised that for Michaelis–Menten kinds of mechanism, any free energy of binding has already been ‘used up’, so the only source of conformational change that will drive the catalytic step comes from thermal fluctuations in both solvent and enzyme.^556,557^ The former necessarily transmits these to the enzyme active site from the protein's surface. Entirely consistent with this, Jiménez-Osés and colleagues^349^ studied two mutants of lovD whose ground state structures were indistinguishable but whose activities varied 1000-fold. Only differences in the dynamics could explain this, and Osuna (*e.g.*, ref. 558–565) and others (*e.g.*, ref. 526 and 566–586) have developed these ideas to exploit the methods of conformational selection and molecular dynamics (MD) to predict which residues might most usefully be mutated in order to navigate the fitnes landscapes more effectively. MD is also of value in the assessment of thermostability.^587–595^ Unsurprisingly, machine learning techniques are now being used in various ways to speed up these kinds of MD calculations.^596–605^

### Modelling methods that effectively draw smooth curves through high-dimensional landscapes

Thus far we have talked about modelling landscapes in rather abstract terms. Classical ‘supervised’ machine learning uses paired properties (here sequence and activity) to learn a mapping that uses the first to predict the second. However, it requires measurements of both sequence and activity that may not always be to hand.^249^ As mentioned above, normally this also requires sequence strings, which are discrete objects, to be converted to a numerical form or ‘embedding’ that allows such a continuous mapping.^548^ The easiest way is arguably to encode each residue with a small vector of numbers that represents its properties (*e.g.*, hydrophobicity, polarity, α-helix-forming tendency, β-sheet-forming tendency, and tendency to be unstructured). This means that biophysically similar amino acids are also close in this vector space.

The breakthrough of the large language models mentioned above was the recognition that much could be learned from sequence alone (known as unsupervised learning), as objects with meaningful structure – whether sentences or images – exhibited statistical regularities. This obviated the need for such large numbers of paired sequences and activity for supervised learning,^606^ and was consistent with the fact that human infants mainly learn languages in an unsupervised way. (For completeness there is also semi-supervised learning, which exploits a smaller number of supervised pairs to improve an otherwise unsupervised model.)

A straight line is of course given by the equation *y* = *mx* + *c*. Thus any of millions of values of *x* can be turned into equivalent values for *y* and if the nature of this curve (line) is known only the two parameters *m* and *c* need be stored. In a similar vein, the idea of this kind of modelling is to find a (complex) equation with a number of parameters far smaller than the number of possible examples (20^*N*^) that effectively plots the entire landscape for millions of sequence examples with which it might be interrogated. Importantly, it was long ago shown that classical artificial feedforward neural networks of the multilayer perceptron^607–611^ or radial basis function^612–614^ types can approximate any function, the so-called universal approximation theorem. Of course this theorem does not precisely state how many parameters are required for a certain degree of approximation,^611^ but the principle is important. More recently, similar arguments have been raised for transformer-based deep neural networks.^615–619^ Thus the first question becomes “can I model a landscape accurately using (deep) neural networks?”, and the answer is yes.

The second question is then, rather obviously, “how many parameters (*i.e.* neural network weights) will I require?”. The answer to this clearly depends on the landscape ruggedness, the efficiency and extent of sampling, the size of the training set, and the size and architecture of the transformer or other approximator. Consequently, there is no universal answer, but there is increasing knowledge of the scaling laws for deep networks: broadly errors decrease exponentially relative to both the number of examples and the number of parameters,^620–623^ and it is best to vary both in step but with substantial data examples per parameter.^624,625^ A useful summary is at https://lifearchitect.ai/chinchilla/.

The reason for this exponentiality actually comes from the bias-variance trade-off in high-dimensional space.^626^ Gratifyingly and importantly, this result fits exactly with the exponential fall off predicted by extreme value theory as rehearsed above, where we seek to fit a population far larger than the small and often biased/local one represented by the samples within the existing population. Consequently interest is shifting towards means of ‘beating’ the standard scaling laws (*e.g.*, ref. 624), that evidently suffer from diminishing returns (not to say massive environmental energy costs).^627^

### Multiobjective optimisation

Thus far we have focused more or less explicitly on single fitness objectives, especially *k*_cat_, but almost all optimisations actually have more than one objective, *e.g.*, specificity (or lack of it), pH dependence, solvent tolerance, and/or, the one we shall focus on, thermostability. Thermostability is desirable not only for reasons of stability but because running reactors at a higher temperature when they contain enzymes with higher values of *k*_cat_ helps avoid cooling costs. Thermostability is also of value when processes are intensive^628,629^ or continuous^506,630–632^ or both.^633^ Obviously reaction rates tend to increase with temperature (for reasons based on basic chemical kinetics, fluctuations, and internal protein mobility given above and by^634^) but protein stability commonly does not.^635^ Consequently there is seen to be some kind of a trade-off^595,636–642^ (also for solubility^643^), although it is not at all obvious that this is inevitable for any kind of fundamental physico-chemical reason.^635^ In fact it is not.

**Fig. 16:**
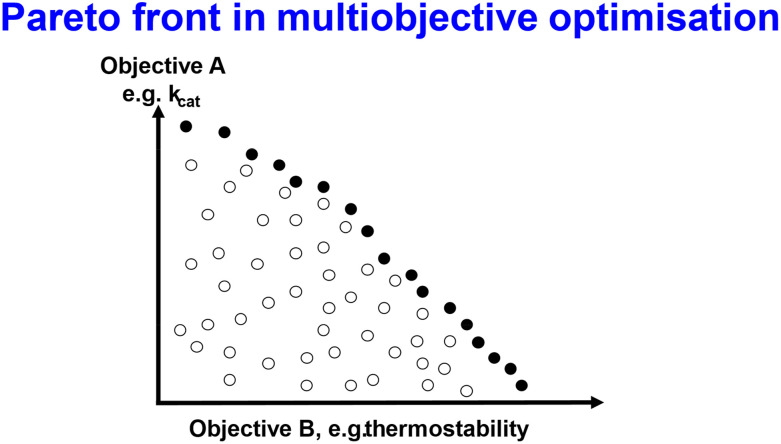
General principle of multiple objectives and the existence of a Pareto front. Individual sequences are represented by symbols, with the filled symbols being on the Pareto or non-dominated front. Redrawn from the CC-By 4.0 Open Access paper.^22^

Fig. 16 illustrates the key general idea of multiobjective optimisation, in which individuals may be especially good at one of the objectives but not the other. Those filled symbols in Fig. 15 represent individuals that are seen as the best in at least one objective for a given value of the other objective, and they occupy what is referred to as the non-dominated or Pareto front. During an adaptive walk it is then a matter of preference as to how (in directed evolution) the experimenter chooses to trade off the two objectives (*e.g.*, ref. 443 and 644). One effective and well-established class of algorithm (*e.g.*, ref. 644–658) seeks to improve individuals by recombining elements of those on the Pareto front in the hope that the Pareto front will effectively move ‘north-east’ in Fig. 15. Memetic algorithms^659–670^ do something similar.

### No necessary trade-off between *k*_cat_ and thermostability

Ancient proteins in an evolutionary sequence tend to be more stable, something considered (assumed) to be reflecting a variety of conditions prevalent at the time,^671^ though they tend to evolve towards more mesophilic or psychrophilic temperature optima and greater catalytic power when the temperature is in the relevant range.^672^ This said, there does not seem to be any *a priori* reason for such a trade-off when sequences are considered more globally and in principle these properties can be decoupled^414,635,641,673–680^ so as to achieve high catalytic activity together with thermostability. Arguably some of the basis for the earlier view was effectively an artefact caused by the fact that many mutations are destabilising so that it appears that activity is in conflict with stability. In addition, comparing thermophilic and mesophilic enzymes at a single temperature erroneously conflates temperature adaptation with activity. Consequently, much as in the spirit of the rest of this review, we consider that the earlier ‘trade-off’ view most likely simply reflects evolutionary contingency, local search, weak mutation/strong selection, and epistasis trapping proteins in local maxima.

Following the multiobjective principle, much as in other areas (*e.g.*, ref. 681) we consider that speeding up directed evolution is best done with a suite of orthogonal methods (including sequence and structural, dynamics, assay-based, and so on). This is because the scientific philosophy principle of coherence^682–685^ states that the more that different and orthogonal methods lead to the same conclusion, the more likely is that conclusion to be correct.

## Discussion and conclusions

### Requests and recommendations

#### The need for a data standard for reporting protein sequence–activity data

Fields such as flow cytometry (https://isac-net.org/page/Data-Standards),^686^ systems biology (https://sbml.org/)^687–689^ and microscopy (https://www.openmicroscopy.org/)^690^ have benefited massively from the existence of data standards in which metadata and observations are reported in a standardised format, produced from any source instrumentation and universally available to any suitable analytical software that consequently only has to be designed to import a specific file type. One thing that became clear during the writing of this review is that the availability of sequence-activity data in an easily accessible and standardised form, whether as spreadsheets or more integrated and formalised formats such as XMLs, was absent. Such a data standard should preferably be modular, ontology-backed, and repository-friendly so creating one seems like an important activity for the Engineering Biology community. The closest is probably the format used by the MAVE (Multiple Analysis of Variance Effects) database MaveDB^161,162^ or EnzymeML.^691^ See also The EnzEngDB (https://enzengdb.org/).^692^ We also note the contrast in metabolomics,^693^ where MetaboLights (https://www.ebi.ac.uk/metabolights/)^694^ and the Metabolomics Workbench (https://www.metabolomicsworkbench.org/)^695,696^ provide data and metadata in standardised and downloadable formats.

We do not think that this is otherwise the place to go into the necessary level of detail, but it does seem that an RO-Crate strategy https://www.researchobject.org/ro-crate/^697^ might be one way forward, while the Analytical Information Markup Language (AniML) (https://www.animl.org/)^698^ would seem to offer the necessary framework for including the necessary data and metadata in a standardised, machine-readable format.

We also note that the Organic Reaction database^699^ bears many similarities to what is desired, while RetroBioCat^700^ is another but far less open reaction database. The Open Reaction Database (ORD) (https://open-reaction-database.org)^699^ is an emerging standard for structuring and sharing organic reaction data in a machine-readable format. The ORD already has some capabilities to include enzymes and proteins as reaction participants, with UniProt ID,^701^ Protein Data Bank,^702^ Hierarchical Editing Language for Macromolecules (HELM) (https://www.pistoiaalliance.org/project/helm-project/), and amino acid sequences being currently supported component identifiers. The ORD is an evolving standard, and the synbio community is encouraged to identify additional data features that may be required for the appropriate description of biocatalytic reactions. Finally, the emergence of ‘vibe coding’ or agentic software^703–705^ that produces code to perform analyses when presented with requests in natural language form offers the oppportunity to democrastise these kinds of analyses.

### Overall conclusions

Existing enzymes selected *via* natural or directed evolution commonly follow a path of weak mutation/strong selection, and the epistatic landscapes that they thereby inhabit necessarily lead to entrapment in local maxima and to ruggedness. The distribution of local fitnesses commonly follows a gamma distribution, but that of the whole landscape is more nearly exponential, indicating that the first is likely to be a poor model for the second. Without models that can cover the landscapes more widely it is hard to extrapolate beyond the known, and active learning is needed to choose wisely the examples with which to populate models that can generalise well to properties far better than those on which they were initially trained. Having such models will potentially usher in an era of enzymes that are designed *de novo*, and are small, thermostable, and highly active. All of these are highly desirable goals.

## Author contributions

Conceptualization, DBK; formal analysis, IR, DBK; resources, DBK; writing – original draft preparation, DBK; writing – review and editing, IR, DBK; visualization, IR, DBK; funding acquisition, DBK.

## Conflicts of interest

There are no conflicts of interest to declare.

## Data Availability

Data used here are in the public domain and their sources full referenced.
